# A randomized controlled trial of physical activity with individual goal-setting and volunteer mentors to overcome sedentary lifestyle in older adults at risk of cognitive decline: the INDIGO trial protocol

**DOI:** 10.1186/s12877-017-0617-y

**Published:** 2017-09-13

**Authors:** Kay L. Cox, Elizabeth V. Cyarto, Christopher Etherton-Beer, Kathryn A. Ellis, Helman Alfonso, Linda Clare, Danny Liew, David Ames, Leon Flicker, Osvaldo P. Almeida, Dina LoGiudice, Nicola T. Lautenschlager

**Affiliations:** 1 School of Medicine, University of Western Australia, Perth, Australia; 2 National Ageing Research Institute, Melbourne, Australia; 3 Royal District Nursing Service (RDNS) Institute, Melbourne, Australia; 4 Academic Unit for Psychiatry of Old Age, Department of Psychiatry, The University of Melbourne, Melbourne, Australia; 5 Western Australian Centre for Health and Ageing, Centre for Medical Research, University of Western Australia, Perth, Australia; 6 Curtin University, Perth, Australia; 7 Centre for Research in Ageing and Cognitive Health, Exeter University, Exeter, UK; 8 School of Public Health and Preventive Medicine, Monash University, Melbourne, Australia; 9 School of Psychiatry and Neurosciences University of Western Australia, Perth, Australia; 10 Melbourne Health, Melbourne, Australia; 11 North Western Mental Health, Melbourne Health, Melbourne, Australia

**Keywords:** Physical activity, Goal-orientated behaviour change, Older adults, Mild cognitive impairment, Peer-mentoring

## Abstract

**Background:**

Increasing physical activity (PA) effectively in those who are inactive is challenging. For those who have subjective memory complaints (SMC) or mild cognitive impairment (MCI) this is a greater challenge necessitating the need for more engaging and innovative approaches. The primary aim of this trial is to determine whether a home-based 6-month PA intervention with individual goal-setting and peer mentors (GM-PA) can significantly increase PA levels in insufficiently active older adults at increased risk of developing Alzheimer’s disease (AD).

**Methods:**

Community living 60–80 year olds with SMC or MCI who do not engage in more than 60 min per week of moderate intensity PA will be recruited from memory clinics and the community via media advertisements to participate in this randomized, single-blind controlled trial. All participants will receive an individually tailored home-based PA program of 150 min of moderate intensity walking/week for 6 months. The intervention group will undertake individual goal-setting and behavioral education workshops with mentor support via telephone (GM-PA). Those randomized to the control group will have standard education workshops and Physical Activity Liaison (PAL) contact via telephone (CO-PA).

Increase in PA is the primary outcome, fitness, cognitive, personality, demographic and clinical parameters will be measured and a health economic analysis performed. A saliva sample will be collected for APOE e4 genotyping. All participants will have a goal-setting interview to determine their PA goals.

Active volunteers aged 50–85 years will be recruited from the community randomized and trained to provide peer support as mentors (intervention group) or PALS (control group) for the 6-month intervention. Mentors and PALS will have PA, exercise self-efficacy and mentoring self-efficacy measured.

Participants in both groups are asked to attend 3 workshops in 6 months. At the first workshop, they will meet their allocated Mentor or PAL who will deliver their respective programs and support via 6 telephone calls during the intervention.

**Discussion:**

If the GM-PA program is successful in increasing the PA levels of the target group it will potentially provide another strategy and community resource that can be translated into practice.

**Trial registration:**

Australia New Zealand Clinical Trials Registry ACTRN12613001181796. (29/10/2013) retrospectively registered.

## Background

In 2015, it was estimated that 46.8 million people worldwide were living with dementia and that this will almost double over the next 20 years [1]. This will impose a significant economic burden on the community. For example, by 2060 in Australia spending on dementia is predicted to overtake any other health condition [2]. Groups at risk for cognitive decline include older adults free of dementia but with Subjective Memory Complaints (SMC) or with Mild Cognitive Impairment (MCI). The prevalence of MCI and SMC increases with age and we have previously shown that it affects 10.6% and 46% of community-dwelling women 70 years and older, respectively [3].

Regular physical activity appears to be one of the strongest factors to delay or prevent cognitive decline [4]. For optimal health benefits, older adults need to perform at least 30 min of moderate-intensity aerobic activity on five days each week [5, 6]. However, unfortunately levels of PA decline with age. For example in Australia only one in three men and one in five women aged 75 years or over undertake sufficient physical activity [7].

Changing sedentary behavior is difficult, with 50% of those starting an exercise program giving up by six months [8, 9]. Groups with health problems face an even greater challenge than healthy individuals [10]. Few studies have investigated strategies that change behavior to initiate the uptake of PA and enhance long-term maintenance in people with SMC and MCI who have an increased risk of AD.

Older adults prefer home-based over center-based physical activity [11]. We have previously shown that a 6-month individualized home-based PA intervention based on the stages of change and social cognitive models increases PA and improves the cognitive function of older adults with SMC and MCI in the short and long-term [12, 13].

In behaviour change research, goal-setting has been used as a method of intervention as well as a measurement tool to assess the efficacy of interventions. Goal-based approaches have been used successively in frail older people [14], people with dementia [15], and healthy older people [16]. The Bangor Goal-Setting Interview (BGSI) [17] allows researchers to select an area of behaviour that is relevant to a study and provides a structure to elicit individual goals and standardise the method of rating the individual’s attainment in relation to the identified goals.

Mentoring has been used in other activities and settings as well as the PA setting to develop skills and change behavior [18–20]. For programs to be successful, older adults need informed advice, and using peer volunteers to deliver programs may improve the translation of evidence-based PA programs [21]. Peer mentors, that is, non-professional individuals who have appropriate training, provide a unique resource to motivate same-age adults [22]. In a study that compared peer-mentoring with counselling by young trained professionals in a structured exercise setting, improvements in perceived physical, mental and social functioning were observed in the peer-mentored group but not with younger mentors [18]. A peer-delivered theory-based advice and support program reported no difference in moderate and vigorous PA after 16 weeks compared to an intervention similar to that offered in the community but after 18 months follow-up, the group with the peer-delivered program had significantly greater increases in moderate and vigorous PA [21]. Further, a telephone-based physical activity intervention delivered by professional staff and peer mentors resulted in similar improvements in physical activity but the peer delivered program was shown to have more versatility and comprehensiveness in the quality of intervention content [23].

Thus peer mentoring has the potential to provide a novel delivery model for PA promotion programs and the dissemination of PA information in the community. There are several questions that need investigation. First, can peer mentors be adequately trained to delivered a theory based intervention in people at increased risk of AD; second, can this be effectively delivered by telephone; third, can peer mentoring be successful in a home-based setting; fourth, will the program lead to greater PA adherence than programs without peer mentoring; and fifth, is the program cost effective?

In addition, little is known about the effect of mentoring on the mentor’s self-efficacy to mentor, their own subsequent PA behavior and well-being. Is the mentoring process beneficial to mentors? Studies on volunteers and counselling have reported improvements in well-being, mental health and self-esteem for counsellors [24, 25]. Furthermore, providing a valued service can lead to an increase in well-being [26]. A novel aspect of this study is that we will evaluate the mentor’s self-efficacy, skills and PA behavior.

The primary aim of this randomized controlled trial (RCT) among insufficiently active older adults at increased risk of developing AD is to determine whether a 6-month home-based physical activity (PA) intervention with individual goal-setting and peer mentors (GM-PA) leads to a significantly greater increase in PA compared to a the same PA program delivered with standard education and peer contact only (CO-PA). We hypothesize that participants randomized to the GM-PA intervention with peer mentors will show a significantly greater increase in their PA at the end of the intervention compared to participants randomized to the control group (CO-PA). A secondary aim is to determine if the GM-PA programs leads to better health outcomes compared to controls. Another secondary aim is to evaluate the health outcomes of peer volunteers.

The Melbourne Health Human Research Ethics Committee approved the study, the participants gave written consent to participate and the project complies with the Declaration of Helsinki.

## Methods/design

### Study design

**Fig. 1:**
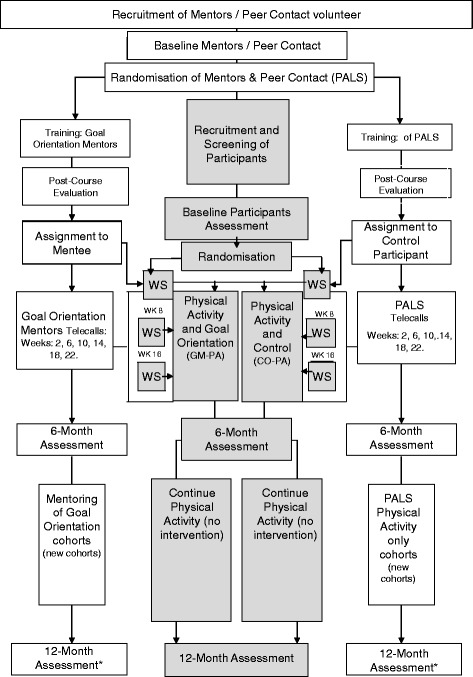
Study Design showing participant flow for the peer mentoring/PALS and study participants (shaded area). WS = Group Workshop. *Mentors and PALs having more than 1 participant will have an additional post intervention assessment at the end of their counselling period

This is a single blind RCT, which is based on CONSORT guidelines (Fig. 1) and includes a health economics analysis. The study has 2 components; (1) the mentoring/peer contact component; (2) the physical activity component.

#### Mentoring and peer contact component

##### Recruitment of peer volunteers

Adults aged 50–85 years meeting the PA guidelines of at least 150 min/week of moderate intensity PA [27] will be recruited from the community via volunteer support groups for seniors and the media in the Melbourne Metropolitan area, We have estimated that we will need to recruit up to 80 peer-age volunteers to provide support for the calculated sample size of study participants. Some peer volunteers may have more than 1 participant over the course of the study. These volunteers will be screened over the telephone with those eligible invited to attend a baseline visit during which written informed consent will be obtained and assessments completed. After baseline assessment volunteers will be randomly allocated to be trained as mentors or peer contacts the latter will be known as physical activity liaisons or PALS.

##### Mentor/PALS outcomes

Volunteers for the mentoring/PALS program will attend a baseline visit during which demographic and lifestyle information will be collected and assessments made of: height; body composition via bio impedance using the Tanita Body Composition Analyzer (Tanita TBF-300, Japan); Body Mass Index (BMI); physical activity assessed with a 7-day pedometer recording; the CHAMPS PA questionnaire [28] and the Stages of Change questionnaire (SOC) [29]; barrier self-efficacy questionnaire (SEQ) [30]; and physical activity specific self-efficacy developed for the INDIGO program (PASSE) using the approach of Ewart and Taylor (1985) [31] (Table 1). These assessments will be repeated at 6 and 12 months for each mentoring/PALS period. Mentoring self-efficacy (MSE) will be assessed from a questionnaire using the same approach as the exercise specific self-efficacy questionnaire [31] but developed around specific mentoring tasks. Both the mentors and PALS will have MSE assessed prior to the training, post training, at the end of their mentee’s/participant’s 6-month intervention period and at 12 months. For mentors or PALS having more than 1 participant they will have an additional post intervention assessment at the end of their counselling period.

**Table 1:** Outcomes and assessment tools for the Mentors and PALs for the INDIGO Study

Assessment Tool	Baseline (0 weeks)	Post-Intervention (24 weeks)	Follow-up^a^ (48 weeks)
Demographic & lifestyle questionnaire	x	x	x
Height, weight, body composition, BMI, girths	x	x	x
Community Healthy Activities Model Program for Seniors (CHAMPS) questionnaire	x	x	x
7-day pedometer records	x	x	x
Stage of Change questionnaire	x	x	x
Barrier Self-Efficacy questionnaire	x	x	x
Physical activity specific Self-Efficacy questionnaire	x	x	x
Mentoring self-efficacy questionnaire (pre & post training course)	x x	x	x
NEO Five-Factor Inventory (NEO-FFI):	x		
Attitudes to Ageing Questionnaire	x	x	x
Training course content evaluation questionnaire	x		
Process evaluation questionnaire		x	x

The X indicates at which point of the trial the respective assessments took place. Follow-up times relate to baseline testing

^a^Mentors and PALs having more than 1 participant will have an additional post intervention assessment at the end of their counselling period

##### Mentor and PAL randomization

After the Mentor/PALS baseline visit volunteers will be randomized to be either a Mentor or PAL using a computer generated list of random numbers with varying block-sizes of 6,8,10 and 12 using the “ralloc” command implemented in Stata 12 statistical [32]. The assignment will be concealed and drawn by a person not directly associated with the conduct of the study.

##### Mentor and physical activity liaison (PALS) training

A training manual, training courses and resources will be developed for the mentors and PALS. The content and conduct of the course, the training manual and the mentoring/PALS processes will be evaluated. Training courses will be ongoing for the first 3 years.

##### Mentor training course

We developed, implemented and evaluated a training program for volunteer older peer mentors in a previous study [33]. This will be used as the basis for the INDIGO study. The 8-h course will cover topics such as roles and responsibilities of the mentor, safe exercise, communication, giving feedback, developing goal setting skills, managing the processes of change and advocacy. Mentors will be given some training and skills (the use of questions and reflective listening) to develop a motivational interviewing style of counseling.

##### PALS training course

Volunteers randomized to the role of PALS will attend a 3-h training course on how to deliver the standard telephone contact to the CO-PA group and their reporting responsibilities. They will have a script to follow and will only ask participants to report the PA program they have been given. PALS will be directed not to engage the participants in any motivational talk about their PA.

##### Allocation of mentors and PALS to participants

Mentors and PALS will be assigned to their respective intervention/control participants using a systematic approach based on sequenced (by identification number) lists of mentor/PALs as well as intervention/control participants.

##### Mentor’s Telephone contact protocol

Mentors will meet their participants at the initial group workshop and arrange mutually convenient telephone contact times with the roles and responsibilities of each of the parties discussed. Over the 6-month intervention period, mentors will make 6 calls at 4-week intervals starting at week 2 then weeks 6, 10, 14, 18 and 22. They will ask participants about their program and how they are going, prompt them to complete and return their PA diaries, engage them in discussion about their relevant goals, give feedback and discuss strategies to keep them on track. There will be a semi-structured script for the mentors focusing on reflective listening, standardization of the mentoring content and format and spontaneous discussion.

##### PALS telephone contact protocol

The PALS will meet with their participants at a workshop and have the same telephone call schedule as described above for the Mentors. These calls will differ in that they will only ask the participants direct questions about their PA program and what they have done and prompt them to complete and return their PA diaries.

##### Mentor and PAL support

Support mechanisms for older volunteers are essential [34]. Both groups will have a senior support person (member of the research team) assigned who will be available for consultation when needed. The senior support person will contact them post workshops and mid-intervention to ensure they stay “on message”.

#### Physical activity component

##### Participants

Insufficiently active (<60 min/week of moderate or vigorous intensity leisure activity), independent-living older participants with memory concerns but without diagnosed dementia will be recruited in the metropolitan area of Melbourne through wide promotion of the project via web sites and newsletters, in clinical settings, via seniors groups and media. The trial will be housed at the National Ageing Research Institute (NARI). Recruitment will be staggered across the first 2 years. Participants defined as having subjective memory complaints (SMC) will need to answer “Yes” to the question “Do you have any difficulty with your memory” and score in the range of normal control group scores for their age and sex on the Cognitive Battery of the Consortium to Establish a Registry for Alzheimer’s Disease (CERAD) [35]. MCI will be defined according to the criteria of Winblad et al. (2004) [36]: (a) memory complaint, (b) evidence of impairment on objective cognitive tasks (measured with a score on the CERAD lower than −1.5 SD or more compared to the norm on any of the CERAD subtest); (c) preserved basic activities of daily living (ADL) and no or only minimal impairment in complex instrumental functions (IADL); (d) does not fulfill DSM-IV criteria for the diagnosis of dementia [37].

### Screening

Volunteers will be screened via telephone by research staff using a screening protocol which worked well for a previous study that recruited similar participants [12]. It will include the Telephone Interview for Cognitive Status – Modified (TICS-M) [38]. Those scoring lower than 19 (out of 50) will be excluded due to the likely presence of dementia. Likewise, individuals with a Geriatric Depression Scale 15 (GDS-15) [39] score of 6 or higher will be excluded due to the presence of clinically relevant symptoms of depression. The Revised Physical Activity Readiness Questionnaire (PAR-Q) [40] which provides information regarding a person’s medical history and any contraindications to exercise will be used to assess whether the participant is eligible to participate in the PA intervention. Those passing the telephone screening will be sent the “Participant Information and Consent Form” and will be required to agree that the research team contact their regular doctor to assure that he/she is happy for the participant to engage in physical activity. Their primary care doctor will be sent a letter describing the study and a form to indicate whether or not their patient should participate - once approved the person will be invited to a baseline visit.

During the screening and the baseline visit participants will be included if they are: aged 60 years or older; community dwelling; and fulfill the study criteria for SMC or MCI (described above). Exclusion criteria include: TICS-M score < 19 [38]; baseline Mini-Mental State Examination score (MMSE) < 24 [41]; diagnosis of dementia; GDS-15 score > 6 [39]; unstable or life threatening medical condition; medical condition that contra-indicates moderate PA; non-sedentary lifestyle (defined as doing more than 60 min/week or more of intentional moderate or higher intensity activity [42]; Body Mass Index (BMI) > 35; severe visual or hearing impairment; history of chronic alcohol abuse within the past five years; and unable to attend the follow-up visits.

### Baseline visit

Participants will sign a written consent form at the baseline assessment. They will also complete a health assessment; provide demographic health and lifestyle information; complete a cognitive and clinical battery, a goal-setting interview and a physical activity and fitness assessment; have a pedometer fitted and give a saliva sample for APOE genotyping (Table 2).

**Table 2:** Outcomes and assessment tools for the participants in the INDIGO Study

Assessment Tool	Baseline (0 weeks)	Post-Intervention (24 weeks)	Follow-up (48 weeks)
Demographic & lifestyle questionnaire	X	X	X
Resting seated blood pressure	X	X	X
Height, weight, body composition, girths	X	X	X
7-day pedometer recording	X	X	X
CHAMPS physical activity questionnaire	X	X	X
Stage of Change questionnaire	X	X	X
Barrier Self-Efficacy questionnaire	X	X	X
Physical Activity Specific Self-Efficacy questionnaire	X	X	X
Balance Step test	X	X	X
Sit-to-stand test	X	X	X
Grip strength test	X	X	X
Timed Up and Go test	X	X	X
Six-minute walk test	X	X	X
Physical activity adherence (monthly diaries)		X	X
Bangor Goal-Setting Interview (BGSI) Modified for INDIGO	X	X	X
Standardized Mini-Mental State Examination (SMME)	X	X	X
Consortium to establish a Registry for Alzheimer’s disease battery (CERAD)	X	X	X
Alzheimer’s disease Assessment Scale 13 – Cognitive section (ADAS-cog 13)	X	X	X
Clinical Dementia Rating Scale (CDR)	X	X	X
Delis-Kaplan Executive Function System (D-KEFS)	X	X	X
Behaviour Rating Inventory of Executive Function – Adult Version (BRIEF-A)	X	X	X
Stroop Task	X	X	X
Digit Symbol Coding	X	X	X
Digit Span (forward and backwards):	X	X	X
Cambridge Contextual Reading Test (CCRT)	X	X	X
Memory Complaint Questionnaire (MAC-Q)	X	X	X
Hospital Anxiety and Depression Scale (HADS)	X	X	X
Short Form-36 version 2 (SF-36v2)	X	X	X
NEO Five-Factor Inventory (NEO-FFI):	X		
Attitudes to Ageing Questionnaire	X	X	X
DNA sample collection	X		
Health Economics questionnaire	X	X	X
Physical Activity Program Evaluation questionnaire		X	X

The X indicates at which point of the trial the respective assessments took place. Follow-up times relate to baseline testing

#### Health assessmenzts

Height will be measured using a fixed stadiometer. Body weight will be measured and body composition will be analyzed with bio impedance using the Tanita Body Composition Analyzer (Tanita TBF-300, Japan). BMI will be calculated in kg/m^2^. Three girths measurements will be taken at the waist and hip using a steel tape (Lufkin, W606 PM Cooper industries SC, USA) and the median measure determined. Resting blood pressure will be measured while sitting after 5 min rest with an automatic blood pressure monitor (A&D UA-767PC (A&D Medical, Thebarton, Australia) 5 times at 2-min intervals. Participants will also be asked to keep a record of illness, medications, injury and any falls.

#### Cognitive and clinical assessment

The cognitive and clinical battery will comprise well-validated psychometric and neuropsychological tests, including tasks sensitive to the presence of mild cognitive impairment and cognitive decline. It will be administered by a trained research assistant at baseline, 6 and 12 months. The research assistant will be blinded to the participant’s group allocation.

##### Standardized mini-mental state examination (SMMSE)

The Mini-Mental State Examination (SMMSE) is a short, commonly used screening instrument of global cognitive function (orientation, attention, memory, language and praxis) with scores ranging from 0 to 30 [41]. The standardized MMSE (SMMSE) [43] applies standardized administration and scoring procedures to the MMSE to improve reliability, and will be used in this study to exclude participants with more severe cognitive impairment and to monitor the cognitive change follow-up.

##### The Consortium to Establish a Registry for Alzheimer’s Disease (CERAD)

The neuropsychological assessment battery from the CERAD [35] is sensitive to early cognitive impairment. The individual tests are: verbal fluency, Boston Naming Test, MMSE, word list and constructional praxis. It will be used to help determine whether participants have MCI at baseline.

##### Alzheimer’s Disease assessment scale 13 – Cognitive section (ADAS-cog 13)

The ADAS-cog [44] widely used to monitor the progression of cognitive deficits in clinical trials consists of a 13-item cognitive battery of short neuropsychological tests. Delayed word recall and number cancellation have been added to the 11-item ADAS-Cog in an effort to boost sensitivity for detecting early cognitive change.

##### Clinical dementia rating scale (CDR)

The CDR is a widely used clinical staging instrument for dementia, useful for globally staging the level of impairment: 0 = No impairment, 0.5, 1, 2, and 3 indicate Very Mild, Mild, Moderate and Severe Dementia, based on a semi-structured interview [45].

##### Behavior rating inventory of executive function – Adult version (BRIEF-A)

The BRIEF-A is a standardized measure that captures views of an adult’s executive functions or self-regulation in his or her everyday environment [46]. The BRIEF-A is composed of 75 self-report items within nine non-overlapping theoretically and empirically derived clinical scales that measure various aspects of executive functioning. These scales include abilities to inhibit, self-monitor, plan/organize, shift, initiate, task monitor, emotional control, working memory, and organization of materials. The BRIEF-A can be completed by a participant outside the testing environment.

##### Short form 36 version 2 (SF-36v2)

The SF-36v2 [47] is a widely used health status questionnaire comprising 36 items which are organized into 8 subscales (Physical Functioning, Role Physical, Bodily Pain, General Health, Vitality, Social Functioning, Role Emotion and Mental Health) from which the Physical and Mental component scales are derived.

##### Memory complaint questionnaire (MAC-Q)

The MAC-Q is a six-item scale of self-reported memory decline in which participants compare current memory ability with past performance for given situations [48]. Scores range from 7 to 35 with higher scores considered to reflect perceived cognitive decline.

##### Hospital anxiety and depression scale (HADS)

The HADS is a 14-item self-rating instrument designed to assess the presence and severity of anxiety and depressive symptoms in medical patients within hospital out-patient, primary-care, and community settings for all age groups [49]. The scale consists of separate seven-item subscales for depression and anxiety.

##### Delis-Kaplan executive function system (D-KEFS) - verbal fluency

The D-KEFS verbal fluency task assesses the participant’s ability to produce verbal responses in accordance with set rules in the time period of 1-min [50]. Letter fluency taps into task initiation, simultaneous processing, systematic retrieval of responses and processing speed. Category fluency requires the same skills as letter fluency, but uses an over learned semantic category as the singular rule (verbal administration only).

##### Stroop task

This test measures cognitive flexibility, gauged by the participant’s effectiveness in processing stimuli as task demands increase over 3 successive trials [51].

##### Digit symbol coding

This subtest of the Wechsler Adult Intelligence Scale (3rd edition) (WAIS–III) [52] measures attention, visual scanning, working memory and speed of information processing.

##### Digit span (forward and backwards)

This WAIS–III subtest [52] will be used to measure attention and working memory.

##### Cambridge contextual reading test (CCRT)

The CCRT [53] sets the National Adult Reading Test (NART) words within semantic and syntactic context, and provides a predicted measure of verbal intelligence.

##### NEO five-factor inventory (NEO-FFI)

This is a 60-item version of the NEO-personality inventory (version 3) that provides a quick, reliable, and accurate measure of the five domains of personality (Neuroticism, Extraversion, Openness, Agreeableness, and Conscientiousness) [54].

##### Attitudes to ageing questionnaire

This is a 24-item cross-cultural attitudes to ageing questionnaire which asks about psychological growth, psychosocial loss and physical changes [55].

#### Physical activity assessment

##### Physical activity measurement

*7-Day Pedometer Recording:* Pedometers will be used to provide an objective measure of PA. Change in steps per day is the primary outcome measure of this study. Participants will be provided with a pedometer (Yamax CW-700/701 Digi-walker, Yamax Co., Japan), and asked to keep to their usual activities and to wear it for five weekdays and the weekend, during the week following the baseline visit. Participants will be instructed on how and when to wear the device, how to record their steps and how much time they spent sitting each day in a 7-day diary. Any time the pedometer is not worn will be recorded and all activities done during this time recorded. The pedometer and diary are posted back to the research team after the measurement period. This measurement will be repeated at follow-up visits.

##### Physical activity questionnaires

*CHAMPS physical activity questionnaire for older adults*: This PA questionnaire, designed for older adults, collects information on various PA, their frequency and duration [28].

*Stage of Change Instrument (SOC)*: The participant’s current stage of PA behavior will be assessed using the SOC [29]. Amount and frequency per week of moderate and vigorous intensity leisure activity will be recorded and used as a measure of moderate and vigorous physical activity (MVPA).

*Self-efficacy Questionnaires:* PA self-efficacy under adverse events (SEQ) will be assessed with a 5-item questionnaire [30]. Program specific PA self-efficacy (PASSE) will be measured using the approach of Ewart and Taylor (1985) [31] where participants rate on a scale of 0–100% their confidence to complete a specific PA task.

#### Functional fitness assessment

Functional fitness will be assessed with a battery of tests briefly outlined below and previously described [56, 57].

*Grip strength*: Measured on both hands with a Smedleys hand dynamometer [58].

*Step Test:* This test assesses dynamic balance. The participant will step one foot on then off a 7.5 cm high step as many times as possible in 15 s without using hand support [59].

*Sit-to-Stand Test*: A test of functional lower limb strength. The participant is seated in a standard chair and stands up and down 5 times as quickly as possible while being timed [60].

*Timed Up and Go Test (TUG):* TUG assesses agility and leg strength. The participant is timed whilst standing up from a standard chair, walking three meters and then returning to sit again in the chair [61].

*6-min Walk Test:* The participant walks as far as possible around a standardized course in 6 min to assess cardiovascular fitness [62]. Heart rate is recorded every minute, peak heart rate determined (Polar FS3c Heart Rate Monitor, Polar Electro Oy, Kempele, Finland) and rate of perceived exertion (RPE) measured at the end of the test [63].

#### Goal identification interview

The Bangor Goal-Setting Interview (BGSI) was developed by Clare & Nelis (2012) [17] to identify goals for goal oriented programs for behavior change in PA and cognitive activity and was based on the Canadian Occupational Performance Measure (COPM) [64]. The interview was adapted for use in the INDIGO study to specifically focus on goals for physical activity. The BGSI will be used to identify PA goals and as an outcome measure of performance and level of satisfaction with current performance. Participants will be asked to discuss the four areas of physical health (PH), physical activity (PA), physical function (PF) and every day function (EDF) and identify any issues that might form the basis of goals. They will rate the importance of and readiness to change in the 2 areas of PF and PA on a scale of 1–10. They will be asked to identify 3–5 goals in the area of PF (e.g., strength, agility, fitness etc.) and PA (type, frequency, duration etc.). The participant will rate the performance and satisfaction for each goal on a scale of 1–10. These goals will be assessed again at the follow-up visits. The use of this new interview will enable us to focus the goal orientation around PA as a motivation for increasing PA in this population.

### Apolipoprotein (APOE) genotyping

As we and others have shown that the APOE genotype can interact with PA outcomes [12, 65] the APOE genotype will be adjusted for in the final analysis. A saliva sample for DNA will be collected at baseline using a Oragene DNA Self-Collection kit (Oragene DNA (OG-500) DNA Genotek Inc., Ontario, Canada). Determination of APOE genotype will be performed centrally at the Biochemistry Department at Royal Perth Hospital, Western Australia, using the method of Hixon and Vrernier (1990) [66]. All samples will be batched and processed once the final baseline assessment has been completed.

### Follow-up assessments

Participants will be contacted 6 and 12 months after baseline. All the cognitive and physical assessments from baseline will be repeated except for the collection of saliva for APOE.

### Program and process evaluation

After 6 and 12 months, participants will be asked to complete questionnaires to give feedback on the program. All participants will receive feedback on their results after their 12 months assessment.

### Randomization and blinding of participants

After the baseline visit participants will be randomised to a goal setting and mentoring intervention group (GM-PA) or control group (CO-PA) according to a list of computer-generated random numbers in varying block sizes using the “ralloc” user-written command implemented in Stata 12 statistical software [32]. This will be done by an investigator not involved with the data collection or intervention and concealed from the study personnel. The research assistants responsible for the cognitive and clinical assessment will be blinded to treatment allocation (single blind). Participants in the intervention group and the control group will have the same number of face-to-face and telephone contacts. The RCT will be a 6 month intervention followed by a further 6-months of continued PA but without further structured encouragement. We have selected this timeframe as we have previously demonstrated that cognitive and health benefits from this level of PA are evident after 6 months [12, 67]. Follow-up after a further 6 months will allow us to examine long term effects of the intervention.

### The physical activity intervention

Given that PA has been found to be beneficial [12] and that everyone should be encouraged to engage in regular PA both groups (GM-PA, CO-PA) will receive the same PA program, an additional 150 min of moderate intensity PA/week. The program is progressive in duration and intensity taking 8 weeks to reach moderate intensity (55–65% heart rate reserve) for each individual. Participants will be asked to complete the PA as 3 × 50-min sessions a week or 5 × 30-min sessions a week. Intensity will be monitored using the Borg Perceived Rate of Exertion scale (RPE 10–12) [63]. They will be encouraged to (although not limited to) complete this as walking to maximise the opportunities for each person to be able to participate within their physical ability, at no financial cost and to reach the target of moderate intensity.

Participants will receive a manual including the same information about progressive walking, water walking, swimming and cycling programs and exercise safety [13]. The PA program will be outlined by a trained facilitator as part of a baseline workshop. They will explain the activities, their frequency, intensity and how to record details in standardised PA diaries. PA diaries will be returned monthly in prepaid envelopes. Participants will receive a report on their PA and fitness at 6 months. They will be asked to continue PA for another 6 months without any contact.

### PA adherence

Adherence to the prescribed PA will be assessed from self-reported PA diaries: type, frequency, duration and intensity of the PA will be determined from these records. Adherence will be calculated as the number of minutes of moderate activity completed relative to the prescribed 150 min/week expressed as a percentage.

### Goal orientation intervention group (GM-PA)

The objective of Goal Orientation program (GO) is to the enhance PA self-efficacy and increase PA. It will be developed from 2 sources. The first is a behavioural intervention package which we previously developed based on the Stages of Change Model and Bandura’s Social Cognitive theory [68]. The package matched to the PA stage of change of the participant focused on the development of self-efficacy using amongst other strategies identification of PA goals and setting short and long-term goals. This approach will be modified to focus on goal orientation and the goal setting techniques described below. Secondly, the goal orientation approach developed by Clare and colleagues [15, 69, 70] and used to improve everyday functioning in early stage AD will be modified for participants with SMC and MCI and used as the basis for the proposed GO intervention. This program will incorporate individual goal identification, goal setting and the development of strategies, practical aids and action plans for goal attainment. The participant’s 5 individual goals will be identified at baseline using the PA and PF information from the BGSI. Participants will be asked to select 3 personal goals from these areas. Examples of these goals could include: increasing PA; getting fitter; improving strength, mobility or balance; or feeling better. These 3 goals will be operationalised and worked on during the intervention period and will be the focus of the GO program.

Participants will receive a resource manual the content of which will be delivered via workshops facilitated by trained research staff and supported by the mentoring program.

### Control group (CO-PA)

In addition to the PA program participants in this group will receive a standard education program including healthy ageing, dealing with stress and depression and enjoying retirement delivered via a resource manual and workshops. They will also receive the standard telephone contact support (described above) during which the volunteer peer-contact (PAL) will follow a script and record progress but not give any feedback or motivational advice.

### Workshop content and delivery

In the first 6 months 3 group-workshops will be conducted for both groups. The first will have a duration of two hours: the first hour will be the same for both groups outlining the PA program, safety, the use of the educational materials, how to record the frequency and intensity of their PA, and the workshop and telephone call schedules and will include a walk session. The GM-PA second hour will focus on identifying specific PA goals, operational goals and strategies that will be employed to attain these goals. For CO-PA the content will be a general health/education topic. The remaining workshops will be of 1-h duration at 8-weekly intervals. GM-PA workshop topics will include: setting SMART goals; evaluating progress; feedback; getting the most out of mentoring; identifying barriers to goal attainment; developing practical aids to overcome specific barriers; reassessment of goals; identification of effective and ineffective strategies and review of the action plan. Participants will be asked to work on the goals, utilise strategies and implement the action plan individually as well as with their mentor in between the workshop sessions. These strategies meet the recommendations for best practice for the maintenance of PA in the long-term [71]. Workshop topics for the CO-PA group include; healthy ageing, stress anxiety and depression, and enjoying retirement. The workshops for the two groups will be held at different times to avoid contamination between the two groups.

### Health economic analysis

Health economic modeling will be employed to estimate the potential cost-effectiveness of GM-PA. Decision analysis [72] will be used to compare the downstream consequences of GM-PA versus CO-PA. The incorporation of Markov [73] and life-tabling [74] techniques will allow for the modelling of outcomes beyond the 12-month duration of the study. The main output of interest is incremental cost-effectiveness ratios in terms of net costs per unit of health gain. Net costs will comprise the costs of GM-PA minus costs saved from the reduction in downstream health services utilization. Health gains will be measured in a variety of ways. Years of life gained and quality-adjusted life years (QALYs) gained will be estimated, both enabled by the collection of time-to-outcome data and quality of life data, as described above. All health economic analyses will be undertaken in accordance with recommended approaches, such as 5% discounting of estimated future costs and health gains. To account for any uncertainty in the data inputs for health economic modeling, sensitivity and uncertainty analyses will be undertaken via Monte Carlo simulation [75].

### Statistical analysis

Participants who withdraw during the trial will be invited to return for the follow-up assessments to allow for an intention-to-treat analysis (primary analysis).

#### Sample size calculation

The primary outcome will be the change in PA at follow-up as measured by the pedometer in steps/day. We have shown that a mean difference of 1200 steps between experimental and control groups would have clinical implications [12]. A study sample size of 53 per group was estimated to give 80% power at α = 0.05 to detect a difference of 1200 steps/day between the groups. Given our previous experience with PA interventions in sedentary older adults we have accounted for a potential 25% attrition rate and also adjusted for the intra-class correlation generated by having one mentor with more than one participant. We calculated the inflation factor 1 + r (c-1), in which, on average, each mentor will have 2 participants (c), and we have assumed an intraclass correlation of 0.15 (r), which is considered of medium size with an estimate of 76 participants per group. For the secondary outcome, quality of life this size sample will also allow us to detect a mean difference between groups of 4.5 standardized units, as measured by the SF-36. We also estimate that this would detect an improvement of 20% in the mentoring self-efficacy scale in mentors with 80% of power at α = 0.05.

#### Data analysis

Continuous variables with normal distribution will be described using means and standard deviations; median and inter-quartile range will be used for those without a normal distribution. Categorical variables will be described using frequency tables. The efficacy of the intervention will be primarily assessed with an intention-to-treat (ITT) analysis at the end of the intervention with secondary analysis for the 12-month time point. This effect will be tested as the interaction between the allocation group (intervention and control) and time, on the primary and secondary outcomes. We will apply multilevel regression models (mixed models) with baseline value of each outcome included in the model as a covariate. In a complementary analysis, we will apply imputation by chained equations to perform an ITT analysis of primary and secondary outcome measures. Alpha will be set at 5% and all statistical tests reported will be two-tailed.

## Discussion

If this study is able to demonstrate that a PA program utilising individual goal-setting and peer mentors leads to a significantly greater increase in PA compared to a the same PA program with standard education and peer contact it will provide evidence that the use of peer mentors to promote the uptake and maintenance of PA in previously insufficiently active older adults at risk of Alzheimer’s disease is both effective and acceptable. Further, it will support the use of the BGSI as both a measurement tool and intervention strategy. Our modified version of the BGSI with a focus on physical activity behavior used in this study will also provide information on the feasibility of its use in a home-based setting with individuals with SMC or MCI who may have additional challenges in initiating the uptake of PA and maintaining the change in PA behavior in the long-term. In addition to the usual barriers to initiating or reaching recommendations for PA for older adults this target group may also experience difficulty in articulating goals, organizing priorities, planning, remembering and carrying out action plans. The structure of the interview around the 4 areas of physical health, physical activity, physical function and every day function with more detailed attention to specific areas of physical activity, physical function and the processes involved as well the reinforcement and support of the mentors on a regular basis will potentially provide a potent strategy for behavior change.

We will also be able to determine whether this change in PA behavior has an effect on other physical, health and cognitive measures. These findings would be highly significant as it has been estimated that 13% of all cases of Alzheimer’s disease are potentially attributable to physical inactivity and that a 10–25% reduction of physical inactivity would prevent between 380,000 to 1 million AD cases worldwide [76]. Currently there are no effective pharmacological strategies to reduce the risk of cognitive decline and AD, resulting in an increasing call to focus on reducing modifiable risk factors such as midlife hypertension, diabetes mellitus, midlife obesity, depression, smoking, cognitive and physical inactivity. It has been estimated that worldwide about one-third of the AD cases might be attributable to potentially modifiable risk factors [77]. The authors also proposed that with a 10% reduction in each of these risk factors per decade by 2050 there would be a reduction in the prevalence of AD by an estimated 8.3%. Targeted, effective and economically viable PA education and intervention strategies will be needed to achieve these reductions. The current intervention has the potential to be such a program.

Attrition rates for PA interventions in older adults of up to 12 months have ranged from 27% to 50% with most of the attrition occurring in the first 3 months [78]. In a 16-week group-based peer mentor delivered PA intervention 17% attrition was reported at 16 weeks but at 18 months this had increased to 62% [21]. This study demonstrated a significant increase in moderate to vigorous intensity increase in the peer- delivered compared to the community program after 18 months. Our study differs from these earlier studies using peer mentors in that it is home-based, has peer support via telephone and employs a PA focused individualized goal-setting measurement tool and behavior change program. Effective strategies for increasing the PA levels of older adults or indeed preventing the increase in sedentary behavior by active older adults [79] that are cost effective and able to be translated into the community are long overdue [21]. Potentially using the resources within the community that are already health or PA focused such as physically active peer mentors is an attractive approach that will improve the cost effectiveness and make this intervention more translatable into community or clinical settings.

To our knowledge this is the first trial to investigate the effects of a goal-orientated home-based PA program with peer mentoring on PA levels in previously inactive older adults with SMC or MCI. Should the main hypothesis be confirmed, we will contribute new knowledge on how to successfully motivate insufficiently active individuals at increased risk of AD to increase their PA. Further this study has been designed to provide training and resources for peer mentors as well as a ready tool for the measurement of PA goals and the implementation of a PA and goal-setting program that could be translated into the community. If the GM-PA program is successful in increasing the PA levels of this target group this program delivered via peer mentors has the potential to provide a cost effective strategy and community resource that can be readily translated into practice.

## Abbreviations

ACTR: Australia New Zealand Clinical Trials Registry ACTR
AD: Alzheimer’s disease
ADAS-cog 13: Alzheimer’s Disease Assessment Scale 13 – cognitive section
ADL: Activities of Daily Living
APOE: Apolipoprotein E Genotyping
BGSI: Bangor Goal-Setting Interview
BMI: Body Mass Index BMI
BRIEF-A: Behavior Rating Inventory of Executive Function – Adult Version
CCRT: Cambridge Contextual Reading Test
CDR: Clinical Dementia Rating Scale
CERAD: Consortium to Establish a Registry for Alzheimer’s Disease
CHAMPS: Community Healthy Activities Model Program for Seniors
CONSORT: Consolidated Standards of reporting trials
CO-PA: control counselling and physical activity program
COPM: Canadian Occupational Performance Measure
D-KEFS: Delis-Kaplan Executive Function System - verbal fluency
DNA: Deoxyribonucleic Acid
DSM-IV: Diagnostic and Statistical Manual of Mental Disorders-IV
EDF: Every Day Function
GDS-15: Geriatric Depression Scale-15
GM-PA: individual goal-setting and peer mentors physical activity program
GO: Goal Orientation
HADS: Hospital Anxiety and Depression Scale
IADL: Instrumental Activities of Daily Living
INDIGO: Individual Goal-setting study
ITT: Intention -To-Treat
MAC-Q: Memory Complaint Questionnaire
MCI: or mild cognitive impairment
MMSE: Mini-Mental State Examination
MSE: Mentoring self-efficacy MSE
MVPA: Moderate and Vigorous Physical Activity
NARI: National Ageing Research Institute
NART: National Adult Reading Test
NEO-FFI: NEO Five-Factor Inventory
PA: physical activity
PAL: Physical Activity Liaison
PAR-Q: Physical Activity Readiness Questionnaire
PASSE: Physical Activity Specific Self-Efficacy questionnaire
PF: Physical Function
PH: Physical Health
RCT: Randomized Controlled Trial
RPE: Rate of Perceived Exertion
SEQ: barrier Self-Efficacy questionnaire
SF-36v2: Short Form 36 Version 2 SF-36v2
SMC: subjective memory complaints
SMMSE: Standardized Mini-Mental State Examination
SOC: Stages of Change questionnaire
TICS-M: Telephone Interview for Cognitive Status – Modified
TUG: Timed Up and Go Test
WAIS–III: Wechsler Adult Intelligence Scale (3rd edition)
